# Self-Position Determination Based on Array Signal Subspace Fitting under Multipath Environments

**DOI:** 10.3390/s23239356

**Published:** 2023-11-23

**Authors:** Zhongkang Cao, Pan Li, Wanghao Tang, Jianfeng Li, Xiaofei Zhang

**Affiliations:** College of Electronic and Information Engineering, Nanjing University of Aeronautics and Astronautics, Nanjing 211106, China; caozhongkang@nuaa.edu.cn (Z.C.); nanhang4yuanlipan@nuaa.edu.cn (P.L.); 17327838568@nuaa.edu.cn (W.T.); zhangxiaofei@nuaa.edu.cn (X.Z.)

**Keywords:** self-position determination, multipath environment, array signal processing, noncooperative signal

## Abstract

A vehicle’s position can be estimated with array receiving signal data without the help of satellite navigation. However, traditional array self-position determination methods are faced with the risk of failure under multipath environments. To deal with this problem, an array signal subspace fitting method is proposed for suppressing the multipath effect. Firstly, all signal incidence angles are estimated with enhanced spatial smoothing and root multiple signal classification (Root-MUSIC). Then, non-line-of-sight (NLOS) components are distinguished from multipath signals using a K-means clustering algorithm. Finally, the signal subspace fitting (SSF) function with a P matrix is established to reduce the NLOS components in multipath signals. Meanwhile, based on the initial clustering estimation, the search area can be significantly reduced, which can lead to less computational complexity. Compared with the C-matrix, oblique projection, initial signal fitting (ISF), multiple signal classification (MUSIC) and signal subspace fitting (SSF), the simulated experiments indicate that the proposed method has better NLOS component suppression performance, less computational complexity and more accurate positioning precision. A numerical analysis shows that the complexity of the proposed method has been reduced by at least 7.64dB. A cumulative distribution function (CDF) analysis demonstrates that the estimation accuracy of the proposed method is increased by 3.10dB compared with the clustering algorithm and 11.77dB compared with MUSIC, ISF and SSF under multipath environments.

## 1. Introduction

A vehicle tends to rely on satellite navigation to determine its location [1]. As the number of satellites in orbit increases, the precision of satellite navigation has greatly improved. However, satellite navigation is susceptible to harsh environments, such as tunnels and urban canyons [2,3,4]. Cooperative vehicle infrastructure systems (CVIS) are widely applied to make up for the shortcomings of satellite positioning [5]. This technology connects all kinds of transportation elements including vehicle clusters, roadside units and wireless network links [6]. Traditional CVIS depends on high-speed cooperative communication, which is constrained by battery capacity and storage resources [7]. The array antenna is widely used in receiving and analyzing noncooperative signals [8]. Self-position determination based on array sensing multiple source data can avoid the communication overhead due to the lack of communication data interaction [9]. The vehicle’s self-position can be estimated using the array signal data fusion method. Self-position awareness based on direct position determination has been discussed in [10], and can achieve accurate estimation for vehicle positioning when signal frequencies are distinguishable. Signal fitting methods are proposed in [11] and achieve better results than multiple signal classification (MUSIC). Nevertheless, these methods only consider the ideal position scenario and often exhibit poor performance under multipath environments [12]. Position determination in the presence of multipath signals has attracted much attention [13,14,15,16,17,18,19]. In the multipath environment, the array sensors usually receive signals propagating along the line-of-sight (LOS) paths and non-line-of-sight (NLOS) paths [20]. The classical super-resolution localization methods are unable to handle the coherent signals resulting from multipath effect unless spatial smoothing technology is applied [21]. However, the application of spatial smoothing technology will introduce changes in signal power, which may cause difficulty in distinguishing between LOS signals and NLOS signals from the perspective of signal strength. A hierarchical clustering architecture is proposed in [22], which allows us to discriminate among possible different interfering scenarios characterized by the same number of jammers via an unsupervised learning clustering fed using a suitable feature set. The clustering algorithm without using a feature set proposed in [23] can associate discrete data to eliminate the fake localization interference [24] and quickly determine the emitter positions in the absence of prior power information, which has potential to solve the fake localization problem under multipath environments.

To avoid a decline in localization accuracy, it is essential that the NLOS information included in multipath signal data is suppressed. The authors of reference [25] introduce a method to eliminate the signal components with power suppression and then derive a C-matrix only containing the necessary signal information to avoid the impact of the interference signals on the estimated results. The oblique projection method presented in [26] can obtain the signal data with specific components. This method completely removes the uninterested components from the raw data via a geometrical insight of the signal space [27]. The above methods all subtract uninterested components from raw data after estimating the original signal form, which may cause incorrect results due to the existence of intermediate estimation errors [28]. Direct localization methods often establish global grid points before searching for target positions [29]. Although accurate position estimation can be obtained with numerous grid points, the computational complexity of dense grid searches increases as more grid points are considered. The adaptive grid-refinement strategy was originally proposed in [30]. The idea behind the grid refinement approach is to start with a coarse grid of locations and then the grid is refined around the estimated locations. This procedure can achieve low computational complexity and fine grid resolution. The grid refinement is improved in [31,32] and more complex refinement approaches are proposed to solve the grid point distribution issue in various global search scenarios. Nevertheless, the grid refinement process needs to iteratively update the grid points, which is time-consuming.

This paper mainly discusses self-position determination under multipath environments and proposes array signal subspace fitting (SSF) for suppressing NLOS components. All measured angles are obtained by means of enhanced spatial smoothing and root multiple signal classification (Root-MUSIC). Then, the vehicle position is initially estimated with the K-means clustering algorithm, and the NLOS components can then be distinguished for each emitter. Next, the cost function of SSF for suppressing multipath effects is directly established using orthogonal projection. Finally, a local grid search around the initial estimation is applied to obtain the precise results instead of using global grid search. Therefore, the proposed method has lower complexity compared with traditional methods. In addition, simulated experiments are carried out to verify that the proposed method has accurate position estimation.

The main contributions of this paper comprise four aspects.

(1) The K-means clustering algorithm is applied to identify NLOS components from the multipath signals with a distance comparison function. The intersection of bearing lines, which is nearest to the adjacent points, is selected as the initial position estimation. The angles formed by the emitters and initial position are considered as reference angles. The distance comparison function is established using the Euclidean distance between the reference angle and DOA estimation results for each emitter.
(2) The SSF cost function for suppressing NLOS components is established to obtain a precise estimation result. The NLOS components of the signal subspace are suppressed with orthogonal projection. The suppressed signal subspace fitting is obtained using the least squares (LS) equation and the orthogonal projection is incorporated into the P matrix in the SSF cost function.
(3) The local grid search of self-position determination is proposed to reduce the computational complexity of the cost function. On the basis of the initial position estimation, the vehicle position is roughly determined. The accurate position determination can be obtained using the cost function calculation on the local grid points distributed around the initial estimation.
(4) The simulation results show that the proposed method has low computational complexity and high position estimation precision. The numerical analysis shows that the computational complexity of the proposed method is at least 7.64dB lower than MUSIC, ISF and SSF. A cumulative distribution function (CDF) analysis demonstrates that 85 percent of the estimated deviation values for the proposed method are 3.10dB smaller than the clustering algorithm and 11.77dB less than MUSIC, ISF and SSF under multipath environments.

Notation:${\left\{\cdot \right\}}^{T}$ and ${\left\{\cdot \right\}}^{H}$ denote the transpose and conjugate transpose, respectively. tr(·), ${∥\cdot ∥}_{2}$ and ${∥\cdot ∥}_{F}$ are the trace, two-norm and Frobenius norm, respectively. $I_{N\times N}$ denotes an *N* × *N* identity matrix and J denotes an anti-identity matrix. E(·) denotes the expectation operator. *j* denotes the imaginary unit. $\hat{\left(\cdot \right)}$ denotes the estimation of $\left(\cdot \right)$. ${\left(\cdot \right)}^{-1}$ is the operator of the inverse matrix. R(a:b,c:d) represents the matrix composed of elements from rows *a* to *b* and columns *c* to *d* from R. R(a:b) represents the matrix composed of elements from rows *a* to *b* from R. $\mathrm{sort}\left(\cdot \right)$ is an operator that arranges elements of $\left(\cdot \right)$ from smallest to largest.

## 2. Signal Model

**Figure 1:**
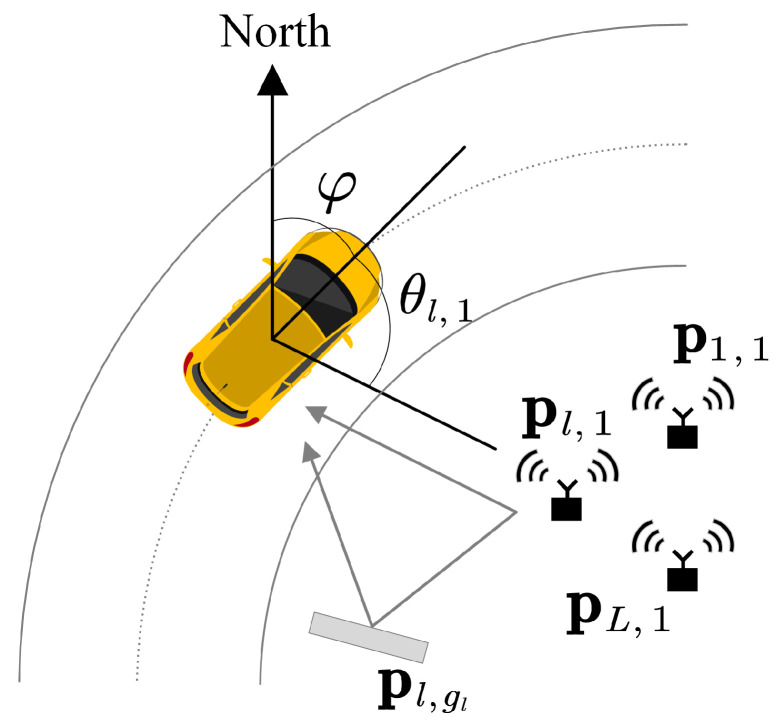
Location scenario.

As is shown in Figure 1, many emitters are distributed around the road, which are, respectively, denoted as $\left\{p_{1,1},p_{2,1},\cdots ,p_{L,1}\right\}$. These emitters radiate signals whose frequencies are distinguishable. The uniform linear array (ULA) with *M* elements is mounted on the vehicle and receives signals radiating from emitters. The array is able to sense the heading angle φ via an electronic compass. The signal incidence angle of the *l*-th emitters is denoted as $\theta_{l,g_{l}}$ relative to the vehicle heading. In practice, there are multiple reflection paths from each emitter to the vehicle. The reflector is denoted as $p_{l,g_{l}}$ related with the *l*-th emitter. The position of the vehicle is represented as $q={\left[q^{x},q^{y}\right]}^{T}$ and the position of the reflector is given as $p_{l,g_{l}}={\left[p_{l,g_{l}}^{x},p_{l,g_{l}}^{y}\right]}^{T}$, where $g_{l}=2,\cdots ,G_{l}$.

The array output data are expressed in the following form:

(1) $$X_{l}\left(t\right)=\sum_{g_{l}=1}^{G_{l}}\left\{\beta_{l,g_{l}}a_{l,g_{l}}\left(\theta_{l,g_{l}}\right)s_{l}\left(t\right)+n_{l,g_{l}}\left(t\right)\right\}=A_{l}S_{l}\left(t\right)+N_{l}\left(t\right)$$

where $\beta_{l,g_{l}}$ is the attenuation coefficient of the $g_{l}$th propagation path of the signal from *l*-th emitter. $a(\theta_{l,g_{l}})$, $s_{l}\left(t\right)$ and $n_{l,g_{l}}\left(t\right)$ are, respectively, the steering vector, the signal source data and the noise source data. $A_{l}$ is defined as the array manifold and satisfies $A_{l}=\left[a\left(\theta_{l,1}\right),a\left(\theta_{l,2}\right),\cdots ,a\left(\theta_{l,G_{l}}\right)\right]$. The signal matrix is written as $S_{l}\left(t\right)$ with $S_{l}\left(t\right)={\left[\beta_{l,1}s_{l}\left(t\right),\beta_{l,2}s_{l}\left(t\right),\cdots ,\beta_{l,G_{l}}s_{l}\left(t\right)\right]}^{T}$. The noise matrix is $N_{l}\left(t\right)={\left[n_{l,1}\left(t\right),n_{l,2}\left(t\right),\cdots ,n_{l,G_{l}}\left(t\right)\right]}^{T}$. The steering vector $a_{l,g_{l}}\left(\theta_{l,g_{l}}\right)$ is defined as

(2) $$a\left(\theta_{l,g_{l}}\right)=\left[\begin{array}{c} e^{j\frac{2\pi }{\lambda_{l}}d\sin\theta_{l,g_{l}}}\\ e^{j\frac{2\pi }{\lambda_{l}}2d\sin\theta_{l,g_{l}}}\\ \vdots \\ e^{j\frac{2\pi }{\lambda_{l}}\left(M-1\right)d\sin\theta_{l,g_{l}}} \end{array}\right]$$

in which *d* denotes the distance between adjacent elements and $\lambda_{l}$ represents the wavelength of signal radiating from the *l*-th emitter.

The array covariance matrix of $X_{l}\left(t\right)$ can be expressed as

(3) $$R_{l}=E\left\{X_{l}\left(t\right)X_{l}^{H}\left(t\right)\right\}=\sum_{t=1}^{T^{l}}\frac{X_{l}\left(t\right)X_{l}^{H}\left(t\right)}{T^{l}}=A_{l}R_{l}^{s}A_{l}^{H}+\sigma_{l}^{2}I_{M\times M}$$

where $R_{l}^{s}=E\left\{S_{l}\left(t\right)S_{l}^{H}\left(t\right)\right\}$ and $E\left\{N_{l}\left(t\right)N_{l}^{H}\left(t\right)\right\}=\sigma_{l}^{2}I_{M\times M}$. $T^{l}$ is the sampling snapshots at one time interval.

The eigenvalue decomposition of $R_{l}$ can be written as

(4) $$R_{l}=U_{l}^{s}\Sigma_{l}^{s}{\left(U_{l}^{s}\right)}^{H}+U_{l}^{n}\Sigma_{l}^{n}{\left(U_{l}^{n}\right)}^{H}$$

where $\Sigma_{l}^{s}$ is the biggest eigenvalue and $\Sigma_{l}^{n}$ is the others. $U_{l}^{s}$ is the signal subspace which consists of the eigenvector corresponding to $\Sigma_{l}^{s}$. $U_{l}^{n}$ is the noise subspace which consists of eigenvectors corresponding to $\Sigma_{l}^{n}$.

The signal subspace can be spanned by the array manifold [11]. So, we can obtain the following equation:

(5) $$U_{l}^{s}=A_{l}T_{l}$$

where $T_{l}$ is a complex coefficient vector.

Self-position determination based on MUSIC, which is proposed in [10], may exhibit the position shift and relative height reduction of spectral peaks due to the influence of multipath signals on noise subspace. So, this paper will establish a cost function that can weaken the multipath effect in Section 3.

## 3. The Proposed Method

### 3.1. DOA Estimation of Multipath Signals

The multipath signal can cause rank deficiency of $R_{l}$, so the enhanced spatial smoothing [33] is adopted to recover the rank of $R_{l}$. Compared with the conventional improved spatial smoothing methods [34,35], the enhanced spatial smoothing can take full advantage of the entire data covariance matrix, therefore significantly improving the decorrelation performance and having stronger noise robustness. The ULA is partitioned into *N* overlapping subarrays, each composed of *K* elements. The enhanced spatial smoothing algorithm can be described as

(6) $$R_{l}^{\mathrm{ESS}}=\frac{1}{2N}\sum_{i=1}^{N}\sum_{j=1}^{N}\left\{\left(R_{l}^{ij}R_{l}^{ji}+{\bar{R}}_{l}^{ij}{\bar{R}}_{l}^{ji}\right)+\left(R_{l}^{ii}R_{l}^{jj}+{\bar{R}}_{l}^{ii}{\bar{R}}_{l}^{jj}\right)\right\}$$

where N=M−K+1, $R_{l}^{ij}=R_{l}\left(\left(i-1\right)K+1:iK,\left(j-1\right)K+1:jK\right)$ and ${\bar{R}}_{l}^{ij}=JR_{l}^{ij}J$.

With the rank recovery covariance matrix, the Root-MUSIC algorithm is applied to estimate the direction of arrival (DOA) values. Similar to Equation (4), $R_{l}^{\mathrm{ESS}}$ can be the eigenvalue decomposed into a noise subspace $U_{l}^{n1}$. Since the noise subspace is orthogonal to the array manifold [36], we can get the 2(K−1)-degree polynomial

(7) $$p_{l}^{T}\left(z^{-1}\right)U_{l}^{n1}{\left(U_{l}^{n1}\right)}^{H}p_{l}\left(z\right)=0$$

where $p_{l}\left(z\right)={\left[1,z,\cdots ,z^{K-1}\right]}^{H}$ and $z=e^{j\frac{2\pi d}{\lambda_{l}}\sin\left(\theta_{l,g_{l}}\right)}$. The roots of Equation (7) are symmetric around the unit circle and the *K* maximum roots inside the unit circle are selected to estimate the DOA results.

(8) $${\hat{\theta }}_{l,g_{l}}=\arcsin\left[\frac{\lambda_{l}}{2\pi d}\arg\left({\hat{z}}_{l,g_{l}}\right)\right]$$

### 3.2. Discrimination of NLOS Components with Clustering Algorithm

The K-means clustering algorithm is a typical unsupervised learning method which is commonly used in object classification [37]. In order to obtain all the possible positioning results, the intersection points of the signal path from different emitters are estimated. The K-means clustering algorithm can identify the center position of different dense point areas by comparing the distance between each point and its surrounding points. In this section, all intersection points between every two bearing lines are calculated. The intersection points from bearing lines of LOS angles $\theta_{1,1},\theta_{2,1},\cdots ,\theta_{L,1}$ tend to densely cluster in an area because the LOS angles are determined by the radiation source positions and the array position. The NLOS angles $\theta_{l,2},\theta_{l,3},\cdots ,\theta_{l,g_{l}}$ are usually determined through random reflector positions and the array position. In the process of clustering, the reflector positions $p_{l,2},p_{l,3},\cdots ,p_{l,G_{l}}$ are assumed to be the corresponding emitter position $p_{l,1}$ so the final intersection positions will be random and cannot point to the unique array position. Therefore, the array position can be determined via finding the center position of the dense point area. To be more explicit, the intersection of the $g_{u}$th bearing lines and the $g_{v}$th bearing lines is defined as $r_{u,v}^{g_{u},g_{v}}=\left[x_{u,v}^{g_{u},g_{v}},y_{u,v}^{g_{u},g_{v}}\right]$ in which u≠v.

(9) $$x_{u,v}^{g_{u},g_{v}}=\frac{p_{u,g_{u}}^{y}-p_{v,g_{v}}^{y}-p_{u,g_{u}}^{x}\tan\left({\hat{\theta }}_{u,g_{u}}+\varphi \right)+p_{v,g_{v}}^{x}\tan\left({\hat{\theta }}_{v,g_{v}}+\varphi \right)}{\tan\left({\hat{\theta }}_{v,g_{v}}+\varphi \right)-\tan\left({\hat{\theta }}_{u,g_{u}}+\varphi \right)}$$

(10) $$y_{u,v}^{g_{u},g_{v}}=\frac{p_{u,g_{u}}^{y}\tan\left({\hat{\theta }}_{v,g_{v}}+\varphi \right)-p_{v,g_{v}}^{y}\tan\left({\hat{\theta }}_{u,g_{u}}+\varphi \right)+\left(p_{v,g_{v}}^{x}-p_{u,g_{u}}^{x}\right)\tan\left({\hat{\theta }}_{u,g_{u}}+\varphi \right)\tan\left({\hat{\theta }}_{v,g_{v}}+\varphi \right)}{\tan\left({\hat{\theta }}_{v,g_{v}}+\varphi \right)-\tan\left({\hat{\theta }}_{u,g_{u}}+\varphi \right)}$$

Based on Equations (9) and (10), the intersection point set can be obtained, which is denoted as $W=\{r_{1,2}^{1,1},r_{1,2}^{1,2},\cdots ,r_{u,v}^{g_{u},g_{v}},\cdots ,r_{L-1,L}^{G_{u},G_{v}}\}$. The *h*th element of W is denoted as $W_{h}$. The distance between $r_{u,v}^{g_{u},g_{v}}$ and $W_{h}$ is defined as

(11) $$d_{u,v,h}^{g_{u},g_{v}}={∥r_{u,v}^{g_{u},g_{v}}-W_{h}∥}_{2},\;where\;W_{h}\neq r_{u,v}^{g_{u},g_{v}}$$

All $d_{u,v,h}^{g_{u},g_{v}}$ derived from $r_{u,v}^{g_{u},g_{v}}$ form a distance set $D_{u,v}^{g_{u},g_{v}}$, the elements of which are sorted in ascending order. A cost function is defined as

(12) $$B_{u,v}^{g_{u},g_{v}}=\sum_{b=1}^{T^{b}}(D_{u,v}^{g_{u},g_{v}}{)}_{b}$$

where $2\leq T^{b}\leq L$ and ${\left(D_{u,v}^{g_{u},g_{v}}\right)}_{b}$ denotes the *b*th element of $D_{u,v}^{g_{u},g_{v}}$. The vehicle position is initially estimated as

(13) $${\hat{q}}_{1}=\min_{r_{u,v}^{g_{u},g_{v}}}B_{u,v}^{g_{u},g_{v}}$$

It is obvious that the bearing line, which is formed by ${\hat{\theta }}_{l}^{\mathrm{LOS}}$ and closest to ${\hat{q}}_{1}$, is the LOS path for each emitter. ${\hat{\theta }}_{l}^{\mathrm{LOS}}$ can be distinguished with the following distance comparison function.

(14) $${\hat{\theta }}_{l}^{\mathrm{LOS}}=\min_{g_{l}=1,2,\cdots ,G_{l}}{∥{\hat{\theta }}_{l,g_{l}}-{\hat{\theta }}_{l}^{q_{1}}∥}_{2}$$

where

(15) $${\hat{\theta }}_{l}^{q_{1}}=\arctan\left(\frac{p_{l,1}^{y}-{\hat{q}}_{1}^{y}}{p_{l,1}^{x}-{\hat{q}}_{1}^{x}}\right)$$

So ${\hat{\theta }}_{l}^{\mathrm{LOS}}$, which is defined as the LOS angle, can be distinguished from $\left\{{\hat{\theta }}_{l,1},{\hat{\theta }}_{l,2},\cdots ,{\hat{\theta }}_{l,G_{l}}\right\}$ and the other angles form the NLOS angle set $\left\{{\hat{\theta }}_{l,1}^{\mathrm{NLOS}},{\hat{\theta }}_{l,2}^{\mathrm{NLOS}},\cdots ,{\hat{\theta }}_{l,G_{l}-1}^{NLOS}\right\}$.

### 3.3. NLOS Data Suppression with Orthogonal Projection

The array manifold of LOS components is written as $A_{l}^{\mathrm{LOS}}$ and the array manifold of others is written as $A_{l}^{\mathrm{NLOS}}$ for the *l*-th emitter. They are defined as

(16) $$A_{l}^{\mathrm{LOS}}=a\left({\hat{\theta }}_{l}^{\mathrm{LOS}}\right)$$

(17) $$A_{l}^{\mathrm{NLOS}}=\left[a\left({\hat{\theta }}_{l,1}^{\mathrm{NLOS}}\right),a\left({\hat{\theta }}_{l,2}^{\mathrm{NLOS}}\right),\cdots ,a\left({\hat{\theta }}_{l,G_{l}-1}^{\mathrm{NLOS}}\right)\right]$$

Equation (5) can be derived as

(18) $$\begin{array}{c} U_{l}^{s}=A_{l}^{\mathrm{LOS}}T_{l}^{\mathrm{LOS}}+A_{l}^{\mathrm{NLOS}}T_{l}^{\mathrm{NLOS}} \end{array}$$

where $T_{l}^{\mathrm{LOS}}\left(t\right)$ and $T_{l}^{\mathrm{NLOS}}\left(t\right)$ are, respectively, the LOS component and NLOS component of $T_{l}$.

To remove the NLOS components, we define the orthogonal projection matrix

(19) $$P_{l}^{⊥}=I_{M\times M}-A_{l}^{\mathrm{NLOS}}{\left({\left(A_{l}^{\mathrm{NLOS}}\right)}^{H}A_{l}^{\mathrm{NLOS}}\right)}^{-1}{\left(A_{l}^{\mathrm{NLOS}}\right)}^{H}$$

which satisfies $P_{l}^{⊥}A_{l}^{\mathrm{NLOS}}=0$. Therefore, Equation (18) can be derived as

(20) $$P_{l}^{⊥}U_{l}^{s}=P_{l}^{⊥}A_{l}^{\mathrm{LOS}}T_{l}^{\mathrm{LOS}}$$

Then, we can obtain the compact data model.

(21) $$P^{⊥}U^{s}=P^{⊥}AT$$

where

(22) $$P^{⊥}=\left[\begin{array}{cccc} P_{1}^{⊥} & \; & & \\ \; & P_{2}^{⊥} & \; & \\ \; & \; & \ddots & \\ \; & \; & & P_{L}^{⊥} \end{array}\right]$$

(23) $$U^{s}={\left[{\left(U_{1}^{s}\right)}^{T},{\left(U_{2}^{s}\right)}^{T},\cdots ,{\left(U_{L}^{s}\right)}^{T}\right]}^{T}$$

(24) $$A=\left[\begin{array}{cccc} A_{1}^{\mathrm{LOS}} & \; & & \\ \; & A_{2}^{\mathrm{LOS}} & \; & \\ \; & \; & \ddots & \\ \; & \; & & A_{L}^{\mathrm{LOS}} \end{array}\right]$$

(25) $$T={\left[\begin{array}{c} T_{1}^{\mathrm{LOS}},T_{2}^{\mathrm{LOS}},\cdots ,T_{L}^{\mathrm{LOS}} \end{array}\right]}^{T}$$

Compared with the data model displayed in Equation (20), the compact data model can greatly describe the correlation of different emitter signals. Therefore, more stable positioning results can be obtained based on Equation (21) due to the application of data correlation. In order to estimate the vehicle position, the SSF cost function with NLOS component suppression is derived in Section 3.4.

### 3.4. Self-Position Determination with Array Signal Subspace Fitting

#### 3.4.1. Grid Search Model

The self-position determination needs uniform grid points in the scenario shown in Figure 1. The distribution of grid points can be assumed to be $X_{m}$ rows and $Y_{m}$ columns. The number of grid points is defined as $Q=X_{m}\times Y_{m}$ and the coordinates of grid points are indexed by $P_{i}={\left[x_{i},y_{i}\right]}^{T}\in R^{2}$, i=1,2,⋯,Q. According to the position relationship between the grid point and the emitter, the characteristic steering vector in the *i*-th grid point can be obtained in the following form:

(26) $$\begin{array}{cc} \phi_{l,i}^{\mathrm{LOS}} & =\left[\begin{array}{c} e^{j\frac{2\pi }{\lambda }d\sin\left({\bar{\theta }}_{l,i}-\varphi \right)}\\ e^{j\frac{2\pi }{\lambda }2d\sin\left({\bar{\theta }}_{l,i}-\varphi \right)}\\ \vdots \\ e^{j\frac{2\pi }{\lambda }\left(M-1\right)d\sin\left({\bar{\theta }}_{l,i}-\varphi \right)} \end{array}\right]\\ \; & =\left[\begin{array}{c} e^{j\frac{2\pi }{\lambda }d\left(\sin\left({\bar{\theta }}_{l,i}\right)\cos\left(\varphi \right)-\cos\left({\bar{\theta }}_{l,i}\right)\sin\left(\varphi \right)\right)}\\ e^{j\frac{2\pi }{\lambda }2d\left(\sin\left({\bar{\theta }}_{l,i}\right)\cos\left(\varphi \right)-\cos\left({\bar{\theta }}_{l,i}\right)\sin\left(\varphi \right)\right)}\\ \vdots \\ e^{j\frac{2\pi }{\lambda }\left(M-1\right)d\left(\sin\left({\bar{\theta }}_{l,i}\right)\cos\left(\varphi \right)-\cos\left({\bar{\theta }}_{l,i}\right)\sin\left(\varphi \right)\right)} \end{array}\right] \end{array}$$

where $\sin({\bar{\theta }}_{l,i})$ satisfies the following relationship:

(27) $$\sin\left({\bar{\theta }}_{l,i}\right)=\frac{p_{l,g_{l}}^{y}-y_{i}}{\sqrt{{\left(p_{l,g_{l}}^{x}-x_{i}\right)}^{2}+{\left(p_{l,g_{l}}^{y}-y_{i}\right)}^{2}}}$$

(28) $$\cos\left({\bar{\theta }}_{l,i}\right)=\frac{p_{l,g_{l}}^{x}-x_{i}}{\sqrt{{\left(p_{l,g_{l}}^{x}-x_{i}\right)}^{2}+{\left(p_{l,g_{l}}^{y}-y_{i}\right)}^{2}}}$$

All the construction matrices are combined into the characteristic array manifold matrix at the *i*-th grid point. The formula is as follows:

(29) $$\Phi_{i}=\left[\begin{array}{cccc} \phi_{1,i}^{\mathrm{LOS}} & \; & & \\ \; & \phi_{2,i}^{\mathrm{LOS}} & \; & \\ \; & \; & \ddots & \\ \; & \; & & \phi_{L,i}^{\mathrm{LOS}} \end{array}\right]$$

#### 3.4.2. Signal Subspace Fitting

On the basis of Equation (21), the following LS equation can be obtained:

(30) $${\hat{q}}_{2},\hat{T}=\min_{i=1,2,\cdots ,Q}{∥P^{⊥}U^{s}-P^{⊥}\Phi_{i}\hat{T}∥}_{F}^{2}$$

$\Phi_{i}$ is assumed as fixed and $\hat{T}$ can be estimated as

(31) $$\hat{T}={\left(\Phi_{i}^{H}{\left(P^{⊥}\right)}^{H}P^{⊥}\Phi_{i}\right)}^{-1}\Phi_{i}^{H}{\left(P^{⊥}\right)}^{H}P^{⊥}U^{s}$$

Define $P={\left(P^{⊥}\right)}^{H}P^{⊥}$. By substituting Equation (31) into Equation (30), the proposed SSF estimator can be derived.

(32) $$\begin{array}{cc} {\hat{q}}_{2} & =\min_{i=1,2,\cdots ,Q}{∥P^{⊥}U^{s}-P^{⊥}\Phi_{i}{\left(\Phi_{i}^{H}{\left(P^{⊥}\right)}^{H}P^{⊥}\Phi_{i}\right)}^{-1}\Phi_{i}^{H}{\left(P^{⊥}\right)}^{H}P^{⊥}U^{s}∥}_{F}^{2}\\ \; & =\min_{i=1,2,\cdots ,Q}\mathrm{tr}\left({\left(U^{s}\right)}^{H}P\left(I_{3M\times 3M}-\Phi_{i}{\left(\Phi_{i}^{H}P\Phi_{i}\right)}^{-1}\Phi_{i}^{H}P\right)U^{s}\right) \end{array}$$

By using the P matrix, the NLOS components in the original signal subspace are suppressed and the SSF estimator can avoid the impact of NLOS components on the estimation results. The vehicle position is the unique variable in Equation (32), so the real position can be accurately determined via calculating the above formula in all grid points. The specific algorithm flow can be seen in Algorithm 1.
**Algorithm 1** Self-Position Determination Based on Array Signal Subspace Fitting under Multipath Environments**Input:** The array receiving data $X_{l}\left(t\right)$,$t=1,2,\cdots ,T^{l}$; The heading angle φ; The emitter position $\left\{p_{1,1},p_{2,1},\cdots ,p_{L,1}\right\}$; The subarray length *K*; The array element number *M*; The vehicle heading angle φ.**Output:** The self-position determination result ${\hat{q}}_{2}$.1:Calculate the array covariance matrix $R_{l}$ from Equation (3);2:Obtain the signal subspace $U_{l}^{s}$ using Equation (4);3:Decorrelate the coherent signal and obtain $R_{l}^{\mathrm{ESS}}$ via Equation (6);4:Obtain the noise subspace $U_{l}^{n1}$ via eigenvalue decomposition similar to Equation (4);5:Estimate the DOA $\left\{{\hat{\theta }}_{1,1},{\hat{\theta }}_{1,2},\cdots ,{\hat{\theta }}_{l,g_{l}},\cdots ,{\hat{\theta }}_{L,G_{L}}\right\}$ with Equations (7) and (8);6:Estimate $r_{u,v}^{g_{u},g_{v}}$ with Equations (9) and (10) for any two bearing lines formed by emitter and corresponding DOA estimation results;7:Calculate $d_{u,v,h}^{g_{u},g_{v}}$ according to Equation (11) and place $d_{u,v,h}^{g_{u},g_{v}}$ in the set $D_{u,v}^{g_{u},g_{v}}$ for each element in $r_{u,v}^{g_{u},g_{v}}$;8:Sort elements of $D_{u,v}^{g_{u},g_{v}}$ in ascending order and obtain $B_{u,v}^{g_{u},g_{v}}$ from Equation (12) for each element in $r_{u,v}^{g_{u},g_{v}}$;9:Estimate the initial position ${\hat{q}}_{1}$ based on Equation (13);10:**for** l=1,2,⋯,L **do**11:The NLOS angles are selected from all estimated DOA results in the *l*-th emitter after considering the angle closest to ${\hat{q}}_{1}$ as LOS angles using Equation (14);12:Construct the orthogonal projection matrix $P_{l}^{⊥}$ with Equation (19);13:**end for**14:Construct $P^{⊥}$, $U^{s}$ and A with Equations (22)–(24);15:Divide the search area into *Q* grid points and construct characteristic array manifold matrices from Equation (26)–(29);16:Calculate Equation (32) and select the grid point with minimum value as ${\hat{q}}_{2}$.

## 4. Performance Analysis

### 4.1. Complexity Analysis

The proposed method consists of seven parts, which are covariance matrix calculation, eigenvalue decomposition, enhanced spatial smoothing, the Root-MUSIC algorithm, the clustering algorithm, orthogonal projection and the SSF estimator. The complexity of the covariance matrix calculation and eigenvalue decomposition are, separately, $LM{\left(T^{l}\right)}^{2}$ and $LM^{3}$. The enhanced spatial smoothing’s complexity is $4LN^{2}K^{3}$ and the Root-MUSIC algorithm’s complexity is ${\left(2K-2\right)}^{3}+2K^{2}+2K-2$. The computational complexity of the clustering algorithm is $\frac{1}{8}{\left(\sum_{u=1}^{L}\sum_{v=1}^{L}G_{u}G_{v}\right)}^{2}+\frac{5}{4}\sum_{u=1}^{L}\sum_{v=1}^{L}G_{u}G_{v}$. The orthogonal projection has a complexity of $\sum_{l=1}^{L}\left\{G_{l}^{3}+3MG_{l}^{2}\right\}$ and the SSF estimator is $27M^{3}+L^{3}+18M^{2}L+6ML^{2}+27M^{2}+LQ$. So, the computational complexity of the proposed method is $O(27M^{3}+LM^{3}+4LN^{2}K^{3}+L^{3}$ $+18M^{2}L+6ML^{2}+27M^{2}+LM{\left(T^{l}\right)}^{2}$ $+LQ+{\left(2K-2\right)}^{3}+2K^{2}+2K-2$ $+\sum_{l=1}^{L}\left\{G_{l}^{3}+3MG_{l}^{2}\right\}+\frac{1}{8}{\left(\sum_{u=1}^{L}\sum_{v=1}^{L}G_{u}G_{v}\right)}^{2}$ $+\frac{5}{4}\sum_{u=1}^{L}\sum_{v=1}^{L}G_{u}G_{v})$.

The complexities for MUSIC [10], ISF [11], SSF [11] and the proposed method are listed in Table 1. The search grid point number of the compared algorithms is denoted as $Q^{\prime }$.

**Figure 2:**
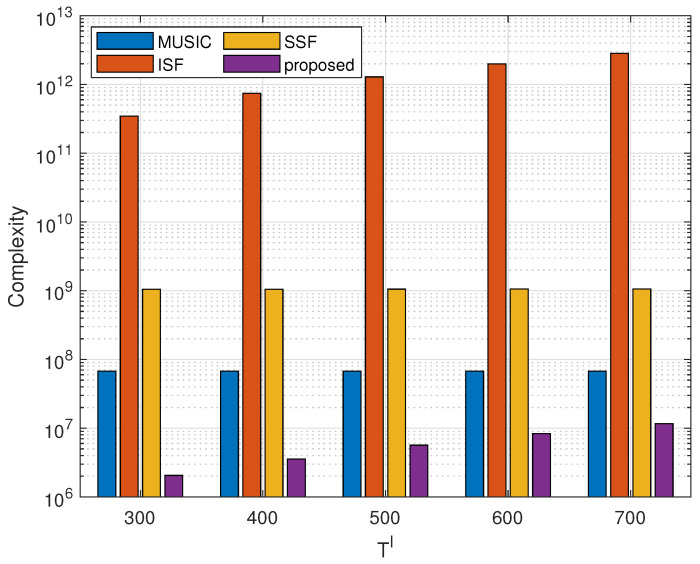
Computational complexity comparison of four methods.

The comparison of computational complexity is shown in Figure 2, where M=10, K=7, N=2, $Q^{\prime }=$ 250,000 and Q= 10,201. The numbers of signal propagation paths are $G_{1}=2$, $G_{2}=2$ and $G_{3}=1$, respectively. The complexity bar chart is increasing with the change of sampling snapshot $T^{l}$. It can be seen that the complexity of the proposed method is at least 7.64dB lower than the others due to the fewer grid points used for accurate estimation after the clustering estimation.

### 4.2. Simulation Results

Several simulated experiments are carried out to verify the effectiveness of the proposed method. Three emitters are distributed in this positioning scenario and two of them each carry one NLOS signal. A vehicle equipped with ULA is considered as a self-positioning target. The emitters are separately located in ${\left[-50,0\right]}^{T}m$, ${\left[-100,250\right]}^{T}m$ and ${\left[-50,500\right]}^{T}m$. The emitters transmit narrowband signals whose frequencies are, respectively, 1000MHz, 1004MHz and 1007MHz. The search area of the compared method is $S_{1}=\left\{{\left[x\;y\right]}^{T}|0\leq x\leq 500,0\leq y\leq 500\right\}$ and the cost function search range of the proposed method is $S_{2}=\left\{{\left[x\;y\right]}^{T}|-50+c\leq x\leq 50+c,-50+c\leq y\leq 50+c\right\}$, where *c* is the initial position estimation result with the clustering algorithm. The grid interval is set as 1m when searching the minimum value of the cost function (Equation (32)). The amplitude attenuation follows this simplified formula:

(33) $$P_{l,g_{l}}^{\prime }=P_{l,g_{l}}^{0}-10\log_{10}∥p_{l,g_{l}}{-q∥}_{2}$$

where $P_{l,g_{l}}^{0}$ denotes the signal radiation power and $P_{l,g_{l}}^{\prime }$ denotes the received signal power. Therefore, the attenuation coefficient $\beta_{l,g_{l}}$ can be defined as

(34) $$\beta_{l,g_{l}}=P_{l,g_{l}}^{\prime }e^{j\alpha_{l,g_{l}}}$$

where $\alpha_{l,g_{l}}$ is the random phase value in the array received signal for the $g_{l}$th path of the *l*-th emitter.

The root mean square error (RMSE) is used to evaluate the precision of the root-MUSIC with enhanced spatial smoothing. The RMSE of the angle estimation is given by

(35) $$\mathrm{RMSE}\left(\theta \right)=\sqrt{\frac{1}{N}\sum_{n=1}^{N}\sum_{l=1}^{L}\sum_{g_{l}=1}^{G_{l}}{∥({\hat{\theta }}_{l,g_{l}}-\theta_{l,g_{l}})∥}_{2}^{2}}$$

where *N* is the Monte Carlo experiment times.

The RMSE values of the root-MUSIC algorithm with enhanced spatial smoothing under different signal-to-noise ratio (SNR) conditions are shown in Table 2, where M=10, K=7, $T^{l}=300$, N=50, $q={\left[251,251\right]}^{T}$ and $P_{l,g_{l}}^{0}=100$. The reflector positions are randomly distributed and SNR varis from 0dB to 25dB. It can be seen from Table 2 that the root-MUSIC algorithm with enhanced spatial smoothing has high-precision angle estimation results under multipath environments.

**Figure 3:**
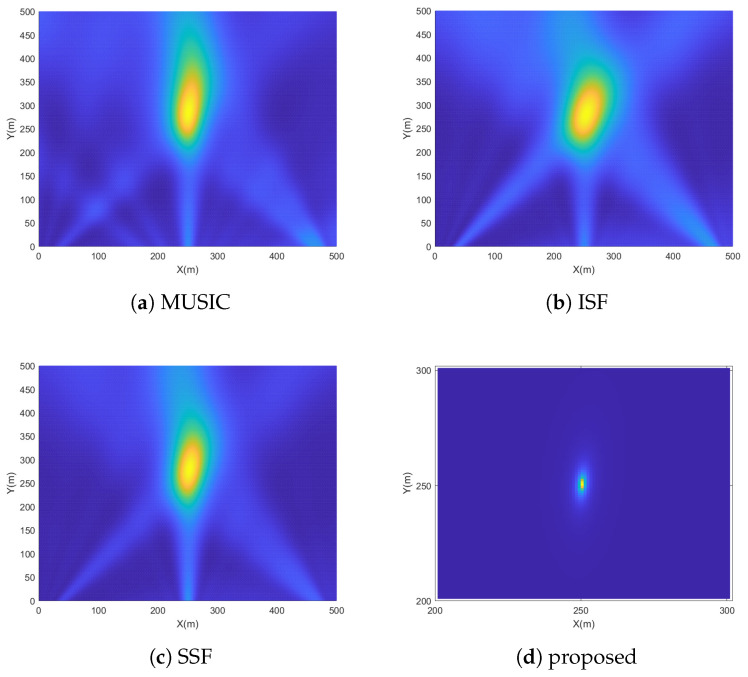
Spectrums of four different methods.

Figure 3 shows the spectrums of MUSIC, ISF, SSF and the proposed method in a simulated experiment at SNR of 10dB, where M=10, K=7, $T^{l}=300$, $T^{b}=2$ and $P_{l,g_{l}}^{0}=100$. For the convenience of comparison, the vehicle position is located in the middle of the search area. It can be clearly observed that the spectral peaks of MUSIC, ISF and SSF deviate significantly from the real position under multipath environments. Due to the NLOS component suppression measures, the spectral peak of the proposed method is sharpest and the position of its maximum value is accurately located near the real position.

**Figure 4:**
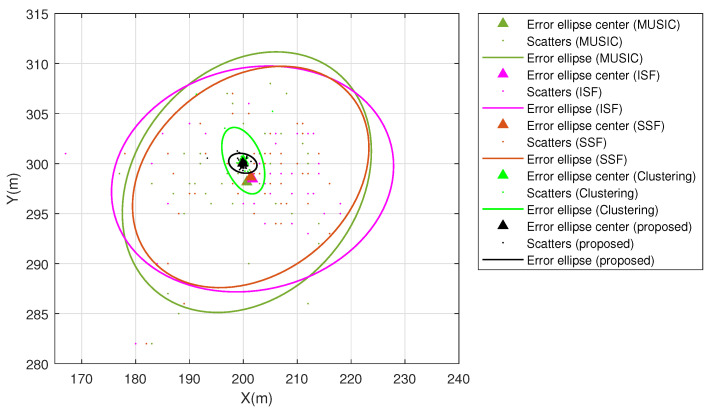
Error ellipse of different methods.

The proposed method is applied to 100 independent Monte Carlo experiments and is compared with MUSIC, ISF, SSF and the clustering algorithm. The vehicle position is fixed at ${\left[200,300\right]}^{T}$. The error ellipses, the confidence region of which is 95%, are employed to describe the accuracy of different algorithms. The simulation results exhibited in Figure 4 indicate that the proposed method has the smallest estimation error range, which is less than 3m. The estimation error range of the clustering algorithm is less than 5m and the estimation error range of MUSIC, ISF and SSF is less than 26m. Moreover, the error ellipse center of the proposed method only has an estimation error of 0.11m, which is less than the clustering algorithm, MUSIC, ISF and SSF. Hence, with the proposed method it is more possible to obtain accurate position estimation than with MUSIC, ISF, SSF and the clustering algorithm.

The definition of RMSE for position estimation is expressed as Equation (36).

(36) $$\mathrm{RMSE}\left(q\right)=\sqrt{\frac{1}{N}\sum_{n=1}^{N}{∥{\hat{q}}_{n}-q∥}_{2}^{2}}$$

where ${\hat{q}}_{n}$ is the estimated position in the *n*th experiment.

**Figure 5:**
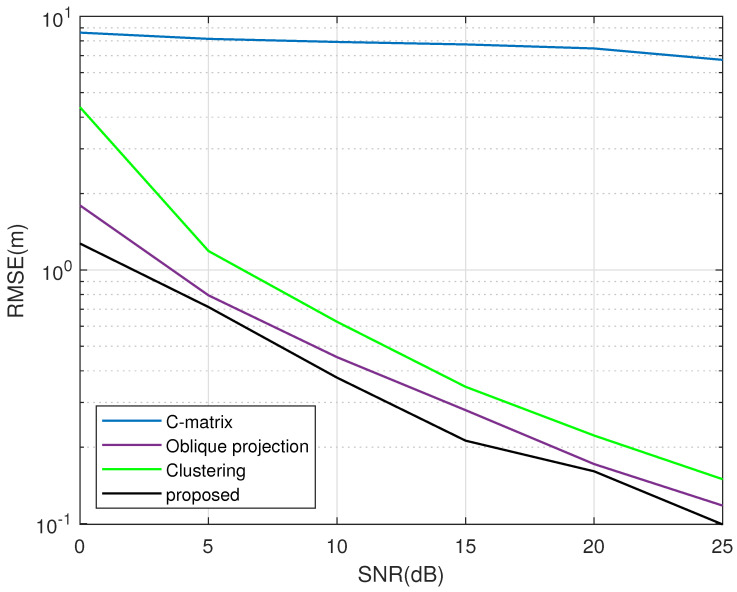
RMSE comparison of different multipath suppression methods.

Figure 5 displays RMSE curves of different multipath suppression methods, where M=15, K=7, $T^{l}=500$, $T^{b}=2$, N=100 and $P_{l,g_{l}}^{0}=100$. The proposed method is compared with C-matrix [25] and oblique projection [26] with respect to the NLOS component’s suppression performance. The simulation results show that the performance of the C-matrix is terrible and its error deviation is even larger than the clustering algorithm. Both the proposed method and oblique projection can greatly reduce the multipath influence. The proposed method can achieve smaller error deviation in comparison with oblique projection.

**Figure 6:**
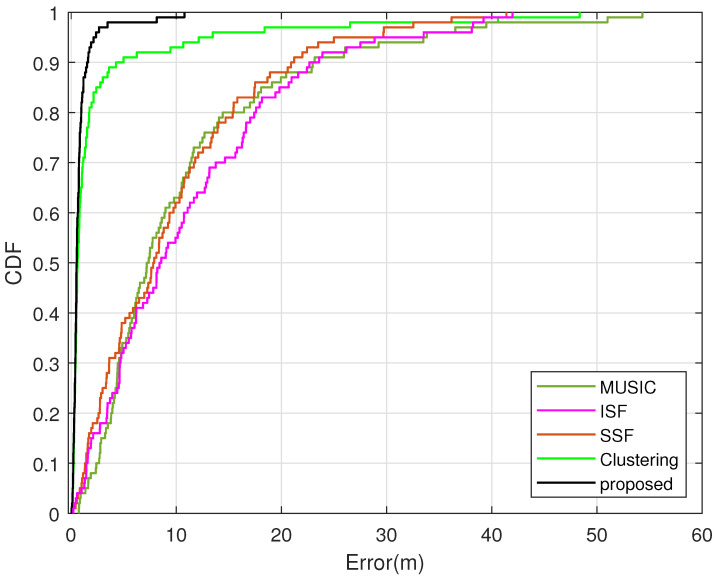
CDF comparison of different methods.

Figure 6 shows the CDF curves versus estimation error, where M=10, K=7, $T^{l}=300$, $T^{b}=2$, N=100 and $P_{l,g_{l}}^{0}=100$. All experiment error values are placed in a set Q whose elements are sorted in ascending order, i.e., $Q=\mathrm{sort}({∥{\hat{q}}_{1}-q∥}_{2},{∥{\hat{q}}_{2}-q∥}_{2},\cdots ,{∥{\hat{q}}_{N}-q∥}_{2})$.

The CDF function is defined as

(37) $$CDF_{Q_{i}}=\frac{i}{N}$$

where *i* is the index of a error value $Q_{i}$ in the set Q.

The vehicle position is randomly set in each simulation process. From Figure 6, it can be seen that the CDF curve of the proposed method is closest to the longitudinal axis and approximately 85 percent of estimation error values are less than 1.16m, which is 3.10dB smaller than the clustering algorithm. Meanwhile, the error deviation of MUSIC, ISF and SSF is much larger and nearly 85 percent of estimation error values are below 17.43m, which is 11.77dB larger than the proposed method. Thus, the proposed method performs with less error deviation than MUSIC, ISF, SSF and the clustering algorithm.

## 5. Conclusions

This paper proposes a self-position determination method based on array signal subspace fitting to suppress NLOS information with a P matrix. The array receiving data are decorrelated via enhanced spatial smoothing and the incident angles are estimated via root-MUSIC. The initial position is estimated using the K-means clustering algorithm and the NLOS components are distinguished with the distance comparison function. The SSF function for suppressing NLOS signal information is directly established, which can obtain the accurate position estimation results. Due to the smaller grid search area, the computational complexity of the proposed method is lower than MUSIC, ISF and SSF via numerical analysis. Further, compared with C-matrix and oblique projection, the proposed method has been proven to perform better in terms of NLOS component suppression performance. Comparisons of spectrums, error ellipses and CDF are carried out to verify the accurate estimation performance of the proposed method.

## Figures and Tables

**Table 1:** Computational complexity of four methods.

Method	Computational Complexity
MUSIC	$O(LM^{3}+LM^{2}T^{L}+LQ^{\prime }M\left(M-1\right))$
ISF	$O(Q^{\prime }L^{3}+6MQ^{\prime }L^{2}+9LQ^{\prime }M^{2}+9Q^{\prime }T^{L}M^{2}+3MQ^{\prime }{\left(T^{L}\right)}^{2})$
SSF	$O(LM^{3}+Q^{\prime }L^{3}+6MQ^{\prime }L^{2}+9LQ^{\prime }M^{2}+LM{\left(T^{l}\right)}^{2}+9Q^{\prime }M^{2}+3MQ^{\prime })$
proposed	$O(27M^{3}+LM^{3}+4LN^{2}K^{3}+L^{3}+18M^{2}L+6ML^{2}+27M^{2}+$ $LM{\left(T^{l}\right)}^{2}+LQ+{\left(2K-2\right)}^{3}+2K^{2}+2K-2+\sum_{l=1}^{L}\{G_{l}^{3}+$ $3MG_{l}^{2}\}+\frac{1}{8}{\left(\sum_{u=1}^{L}\sum_{v=1}^{L}G_{u}G_{v}\right)}^{2}+\frac{5}{4}\sum_{u=1}^{L}\sum_{v=1}^{L}G_{u}G_{v})$

**Table 2:** RMSE of root-MUSIC algorithm with enhanced spatial smoothing under different SNR conditions.

SNR	RMSE (${}^{\circ }$)
0 dB	0.3945
5 dB	0.2514
10 dB	0.1513
15 dB	0.1132
20 dB	0.0464
25 dB	0.0250

## Data Availability

Data are contained within the article.

## Abbreviations

The following abbreviations are used in this manuscript:

CVIS	Cooperative Vehicle Infrastructure Systems
SSF	Signal Subspace Fitting
ISF	Initial Signal Fitting
NLOS	Non-Line-Of-Sight
LOS	Line-Of-Sight
ULA	Uniform Linear Array
DOA	Direction Of Arrival
MUSIC	Multiple Signal Classification
LS	Least Squares
SNR	Signal-To-Noise Ratio
CDF	Cumulative Distribution Function
RMSE	Root Mean Square Error

## References

[1] Jia M., Khalife J., Kassas Z.M. (2023). Performance Analysis of Opportunistic ARAIM for Navigation with GNSS Signals Fused with Terrestrial Signals of Opportunity. IEEE Trans. Intell. Transp. Syst..

[2] Zhu J., Zhou H., Wang Z., Yang S. (2023). Improved Multi-Sensor Fusion Positioning System Based on GNSS/LiDAR/Vision/IMU with Semi-Tight Coupling and Graph Optimization in GNSS Challenging Environments. IEEE Access.

[3] Tang C., Wang Y., Zhang L., Zhang Y. (2022). GNSS/Inertial Navigation/Wireless Station Fusion UAV 3-D Positioning Algorithm with Urban Canyon Environment. IEEE Sens. J..

[4] Sun Y., Cao L., Li S., Deng Z. (2023). G5GIM: Integrity Monitoring for GNSS/5G Integrated Navigation of Urban Vehicles. IEEE Trans. Instrum. Meas..

[5] Zhang P., Tian D., Zhou J., Duan X., Sheng Z., Zhao D., Cao D. (2023). Joint Optimization of Platoon Control and Resource Scheduling in Cooperative Vehicle-Infrastructure System. IEEE Trans. Intell. Veh..

[6] Wang J., Shao Y., Ge Y., Yu R. (2019). A Survey of Vehicle to Everything (V2X) Testing. Sensors.

[7] Han X., Tian D., Sheng Z., Duan X., Zhou J., Hao W., Long K., Xhen M., Leung C.M. (2020). Reliability-Aware Joint Optimization for Cooperative Vehicular Communication and Computing. IEEE Trans. Intell. Transp. Syst..

[8] Gan L., Jiang W., Chen Q., Li X., Zhou Z., Gong S. (2021). Method to Estimate Antenna Mode Radar Cross Section of Large-Scale Array Antennas. IEEE Trans. Antennas Propag..

[9] Yao J., Zhao C., Bai J., Ren Y., Wang Y., Miao J. (2023). Satellite Interference Source Direction of Arrival (DOA) Estimation Based on Frequency Domain Covariance Matrix Reconstruction. Sensors.

[10] Li J., Li P., Li P., Tang L., Zhang X., Wu Q. (2022). Self-Position Awareness Based on Cascade Direct Localization over Multiple Source Data. IEEE Trans. Intell. Transp. Syst..

[11] Cao Z., Li P., Li J., Zhang X., Wu Q. Direct Self-Position Awareness Based on Array-Sensing Multiple Source Data Fitting. Proceedings of the 2023 4th Information Communication Technologies Conference (ICTC).

[12] Hao K., Wan Q. (2021). Sparse Bayesian Inference-Based Direct Off-Grid Position Determination in Multipath Environments. IEEE Wirel. Commun. Lett..

[13] Zhang L., Chen M., Wang X., Wang Z. (2019). TOA Estimation of Chirp Signal in Dense Multipath Environment for Low-Cost Acoustic Ranging. IEEE Trans. Instrum. Meas..

[14] Liu Y., Tan Z.-W., Khong A.W.H., Liu H. (2023). An Iterative Implementation-Based Approach for Joint Source Localization and Association Under Multipath Propagation Environments. IEEE Trans. Signal Process..

[15] Van Marter J.P., Dabak A.G., Al-Dhahir N., Torlak M. (2023). Support Vector Regression for Bluetooth Ranging in Multipath Environments. IEEE Internet Things J..

[16] Aubry A., De Maio A., Foglia G., Orlando D. (2015). Diffuse Multipath Exploitation for Adaptive Radar Detection. IEEE Trans. Signal Process..

[17] Hayvaci H.T., De Maio A., Erricolo D. Diversity in Receiving Strategies Based on Time-Delay Analysis in the Presence of Multipath. Proceedings of the 2011 IEEE RadarCon (RADAR).

[18] Hayvaci H.T., De Maio A., Erricolo D. Performance Analysis of Diverse GLRT Detectors in the Presence of Multipath. Proceedings of the 2012 IEEE Radar Conference.

[19] Rong Y., Aubry A., De Maio A., Tang M. (2020). Diffuse Multipath Exploitation for Adaptive Detection of Range Distributed Targets. IEEE Trans. Signal Process..

[20] Dun H., Tiberius C.C.J.M., Janssen G.J.M. (2020). Positioning in a Multipath Channel Using OFDM Signals with Carrier Phase Tracking. IEEE Access.

[21] Yang Z., Stoica P., Tang J. (2019). Source Resolvability of Spatial-Smoothing-Based Subspace Methods: A Hadamard Product Perspective. IEEE Trans. Signal Process..

[22] Carotenuto V., De Maio A. (2021). A Clustering Approach for Jamming Environment Classification. IEEE Trans. Aerosp. Electron. Syst..

[23] Li J., He Y., Zhang X., Wu Q. (2021). Simultaneous Localization of Multiple Unknown Emitters Based on UAV Monitoring Big Data. IEEE Trans. Ind. Inform..

[24] Guo X., Chen Z., Hu X., Li X. (2019). Multi-Source Localization Using Time of Arrival Self-Clustering Method in Wireless Sensor Networks. IEEE Access.

[25] Zhang Y., Ye Z. (2008). Efficient Method of DOA Estimation for Uncorrelated and Coherent Signals. IEEE Antennas Wirel. Propag. Lett..

[26] Xu X., Ye Z., Zhang Y., Chang C. (2006). A Deflation Approach to Direction of Arrival Estimation for Symmetric Uniform Linear Array. IEEE Antennas Wirel. Propag. Lett..

[27] Zhang X., He Z., Liao B., Yang Y., Zhang J., Zhang X. (2019). Flexible Array Response Control via Oblique Projection. IEEE Trans. Signal Process..

[28] Tao H., Xin J., Wang J., Zheng N., Sano A. (2015). Two-Dimensional Direction Estimation for a Mixture of Noncoherent and Coherent Signals. IEEE Trans. Signal Process..

[29] Tirer T., Weiss A.J. (2016). High Resolution Direct Position Determination of Radio Frequency Sources. IEEE Signal Process. Lett..

[30] Malioutov D., Cetin M., Willsky A.S. (2005). A Sparse Signal Reconstruction Perspective for Source Localization with Sensor Arrays. IEEE Trans. Signal Process..

[31] Hyder M.M., Mahata K. (2010). Direction-of-Arrival Estimation Using a Mixed *ℓ*_2,0_ Norm Approximation. IEEE Trans. Signal Process..

[32] Garcia N., Wymeersch H., Larsson E.G., Haimovich A.M., Coulon M. (2017). Direct Localization for Massive MIMO. IEEE Trans. Signal Process..

[33] Pan J., Sun M., Wang Y., Zhang X. (2020). An Enhanced Spatial Smoothing Technique with ESPRIT Algorithm for Direction of Arrival Estimation in Coherent Scenarios. IEEE Trans. Signal Process..

[34] Du W., Kirlin R.L. (1991). Improved Spatial Smoothing Techniques for DOA Estimation of Coherent Signals. IEEE Trans. Signal Process..

[35] Dong M., Zhang S., Wu X., Zhang H. A High Resolution Spatial Smoothing Algorithm. Proceedings of the 2007 International Symposium on Microwave, Antenna, Propagation and EMC Technologies for Wireless Communications.

[36] Zhu Y., Zhang W., Yi H., Xu H. (2023). Enhanced Root-MUSIC Algorithm Based on Matrix Reconstruction for Frequency Estimation. Sensors.

[37] Uykan Z. (2023). Fusion of Centroid-Based Clustering with Graph Clustering: An Expectation-Maximization-Based Hybrid Clustering. IEEE Trans. Neural Netw. Learn. Syst..

